# Physician perception regarding side-effect profile at the onset of antidepressant treatment: a survey of Israeli psychiatrists and primary care physicians

**DOI:** 10.1186/s12991-016-0090-6

**Published:** 2016-01-29

**Authors:** Uri Nitzan, Tal Bekerman, Gideon Becker, Pesach Lichtenberg, Shaul Lev-Ran, Garry Walter, Hagai Maoz, Yuval Bloch

**Affiliations:** Shalvata Mental Health Care Center, 13 Aliyat Hanoar St, 45100 Hod-Hasharon, Israel; Sackler School of Medicine, Tel Aviv University, Ramat Aviv, Tel Aviv, Israel; Herzog Hospital, Givat Shaul, Jerusalem, Israel; Faculty of Medicine, Hebrew University, Jerusalem, Israel; Addiction Medicine Services, Department of Psychiatry, Sheba Medical Center, Tel-Hashomer, Ramat Gan, Tel Aviv, Israel; Discipline of Psychiatry, University of Sydney, Sydney, NSW Australia; Northern Sydney Local Health District, Sydney, Australia

**Keywords:** Compliance, Adherence, Antidepressants, SSRIs, Side effects

## Abstract

**Background:**

One of the major factors affecting treatment compliance and outcome in patients is the wide range of side effects (SEs) associated with antidepressants. In the present study, we aimed to assess the extent to which Israeli primary care (PC) physicians and psychiatrists discuss the SEs of selective serotonin reuptake inhibitors (SSRIs) with patients prior to the onset of treatment.

**Methods:**

A cross-sectional questionnaire survey was conducted among PC physicians (*N* = 123) and psychiatrists (*N* = 105). Questionnaires were distributed using a mixed-modality design, combining a web survey and in-person delivery of questionnaires.

**Results:**

A significant percentage of our respondents reported that they rarely discuss psychological (60 %) or severe (29 %) SEs of SSRIs. Nearly half (41 %) admitted to avoiding discussion of impact on suicidal ideation. Specialists were noted to discuss and evaluate SEs significantly more than residents, and Psychiatrists more than PC physicians. Specifically, psychiatrists more often discussed the possibility of sexual dysfunction (*t* (225) = 2.23; *p* < 0.05) and suicidal ideation (*t* (225) = 2.11; *p* < 0.05).

**Conclusions:**

It seems that PC physicians and psychiatrists surveyed in this study do not share sufficient information regarding the SEs of SSRIs with their patients at the onset of treatment. In improving this practice, the integration of proper SE management into educational interventions has potential in enhancing compliance and improving expertise and level of care.

## Background

Over the last three decades, prescribing of selective serotonin reuptake inhibitors (SSRIs) has increased dramatically. Awareness regarding the high prevalence of depression and other psychopathologies has grown, and the use of medication has become commonplace [1–3]. Unfortunately, patients seem to be unsure whether they receive adequate information regarding their antidepressant [4] and many believe they would benefit from additional information tailored to their personal needs [5, 6]. Among patients, one of the major factors affecting compliance levels and treatment outcome is the wide range of side effects (SEs) associated with SSRIs [7–9].

SSRIs, for the most part, do not demand a complicated prescription regimen according to accepted guidelines [10, 11]. They present a rather similar than dissimilar efficacy, but certainly they differ in the profile and prevalence of SEs. Thus, choosing an SSRI for a specific patient depends largely on anticipated SEs [12]. Evidence supports the role of shared decision making in enhancing compliance, and more specifically discussing SEs with the patient in advance [13–15]. Beyond the clinical and ethical considerations that pertain to patients in providing informed consent to therapy, the discussion of SEs is effectively a measurable and learnable skill and a core facet of the physician’s expertise [9]. Nonetheless, it is unclear how the provision of information regarding SEs is implemented in clinical practice. In a search of the available literature online, we could not find a systematic attempt to evaluate the extent and quality of the discussion regarding SEs prior to the initiation of antidepressants. Thus, the aim of this study was to assess the extent to which PC physicians and psychiatrist discuss the SEs of SSRIs with their patients and subsequently evaluate them.

## Methods

### Study design

We conducted a cross-sectional questionnaire survey among Israeli PC physicians and psychiatrists. Questionnaires were distributed using a convenient mixed-modal design, combining both web survey and in-person delivery of questionnaires. First, emails were sent with a link to the web-based questionnaire. The emails were sent via mailing lists of the psychiatrists working in the outpatient clinics of six main psychiatric hospitals, located in various areas of Israel. Additional emails were sent to family physicians working in the greater Tel Aviv Metropolitan Area. Questionnaires were also distributed in person, and participants were asked to return them unanswered in case they had already completed the online version. The study was approved by the Institutional Review Board at our facility.

### The questionnaire

A 32-item questionnaire was composed specifically for this study by a panel of five senior psychiatrists and an expert in designing survey (available online at: http://hospitals.clalit.co.il/hospitals/Shalvata/he-il/ArticlesAndResearch/Documents/selfreportquestionnarie.pdf).

The questionnaire was designed to assess the frequency and extent of discussing different groups of SEs with patients prior to the initial prescription of SSRIs: Commonly investigated SEs included headache, sexual functioning, and GI (gastrointestinal) symptoms; severe SEs such as hyponatremia, agitation, and suicidal ideation; and psychological SEs such as emotional bluntness, anxiety, and crying-fits. In addition, the questionnaire assessed the actual number of SEs addressed by the physician, whether the discussion was tailored to the patient, and the nature of the follow-up plan. Socio-demographic data were also collected, including age, gender, profession, seniority, work experience, main place of work, and number of patients evaluated and prescribed SSRI prescriptions per month. In addition, an exploratory factor analysis with varimax rotation was conducted yielding one major factor (Eigenvalue = 6.86). No other significant factors were found.

The questionnaire included a scale in which respondents rated their level of agreement with each statement contained therein on a scale from 1, never to 5, always. When questions related to the actual numbers of SEs, participants had to choose 1 out of 5 categories ranging between 0 and >7. An Expert Explanation Score (EES) was calculated as the average score of fifteen questions (7 to 21) that directly address the explanation and evaluation of SEs with possible scores ranging from 1 to 5. A score of 1 meant that there was an infrequent and/or spotty discussion of SEs, while a score of 5 indicated a detailed and/or frequent discussion and evaluation of SEs. Cronbach’s alpha for the EES was 0.851.

### Data analysis

Statistical analysis was conducted with SPSS version 19.0 for Windows. Differences in proportion of demographic variables were conducted using the Chi-square test. Comparison of groups regarding questionnaire score was done by one-way ANOVA, using independent sample *t* test for some specific comparisons. Significant level was set at *p* < 0.05. Additional analysis was done using Pearson’s correlation.

## Results

A total of 316 physicians were contacted via both modalities, of whom 228 completed the survey (response rate of 72 %); among them were 105 psychiatrists (43 specialists and 62 residents) and 123 PC physicians (28 specialists and 95 residents). The online questionnaire was answered by 141 participants, while 87 completed the hard copy. Among participants who received the questionnaire in person, only two stated that they had already responded to the online version. No significant differences in results were found between questionnaires completed via internet and those administered in person.

Age of the surveyed populations ranged from 28 to 70 years, with a mean age of 43.71 (SD = 10.52). No significant socio-demographic differences were found between PC physicians and psychiatrists in terms of age (*t*(220) = 0.51; *p* > 0.05) and gender (*X*^*2*^ = 2.03; *p* > 0.05). Seventy-five percent of PC physicians reported administering SSRIS to <5 patients a month, whereas over 80 % of psychiatrists reported administering SSRIs to 5–15 patients per month. Socio-demographic variables of the respondents are presented in Table 1.

**Table 1:** Socio-demographic statistics of PC physicians (*N* = 123) and Psychiatrists (*N* = 105)

		Psychiatrists	PC physicians
Gender	Male	50 (48 %)	48 (38 %)
	Female	55 (52 %)	77 (62 %)
Status	Resident	43 (41 %)	28 (23 %)
	Specialists	62 (59 %)	95 (77 %)
Seniority	Up to 5 years	46 (44 %)	35 (28 %)
	Between 6–10 years	16 (15 %)	28 (23 %)
	10 years and more	43 (41 %)	60 (49 %)
Place of work^a^	Open psych department	18 (19 %)	4 (4 %)
	Closed psych department	13 (13 %)	2 (2 %)
	Hospital clinic	21 (22 %)	1 (1 %)
	Community clinic	35 (38 %)	94 (81 %)
	Private clinic	9 (8 %)	14 (12 %)

^a^Nine psychiatrics and eight PC physicians did not answer this question

**Fig. 1:**
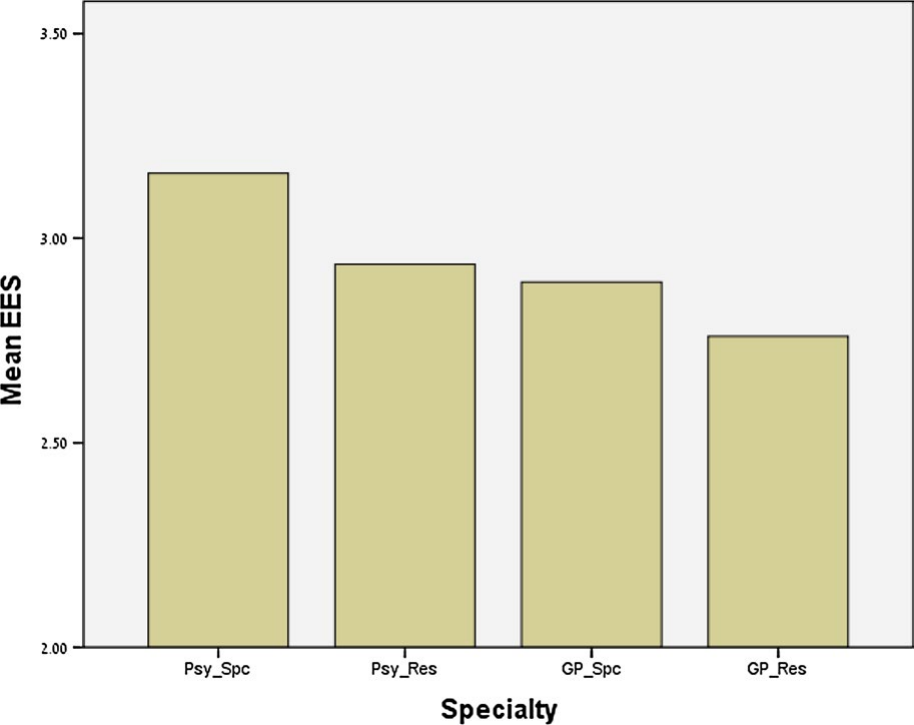
Mean Expert Explanation Score (EES) among senior psychiatrists (Psy_Spc), psychiatry residents (Psy_Res), Senior PC physicians (GP_Spc), and PC residents (GP_Res)

The one-way ANOVA revealed a significant difference amongst groups in the EES (*F*(3224) = 6.09; *p* < 0.01). Additional polynomial contrasts revealed a significant linear pattern (*F*(1224) = 5.14; *p* < 0.05) showing higher EES among Psychiatry specialists, followed by psychiatry residents, PC specialists, and finally PC residents, who achieved the lowest score (Fig. 1).

Twenty-nine percent of the participants in the study seldom or never discussed severe SEs of SSRIs with their patients (26 % of psychiatrists and 30 % of PC physicians), while 60 % of the physicians did not discuss psychological SEs of SSRIs (58 % of psychiatrists and 62 % of PC physicians), and 40 % seldom or never discussed suicide ideation related to SSRIs (36 % of psychiatrists and 43 % of PC physicians).

**Fig. 2:**
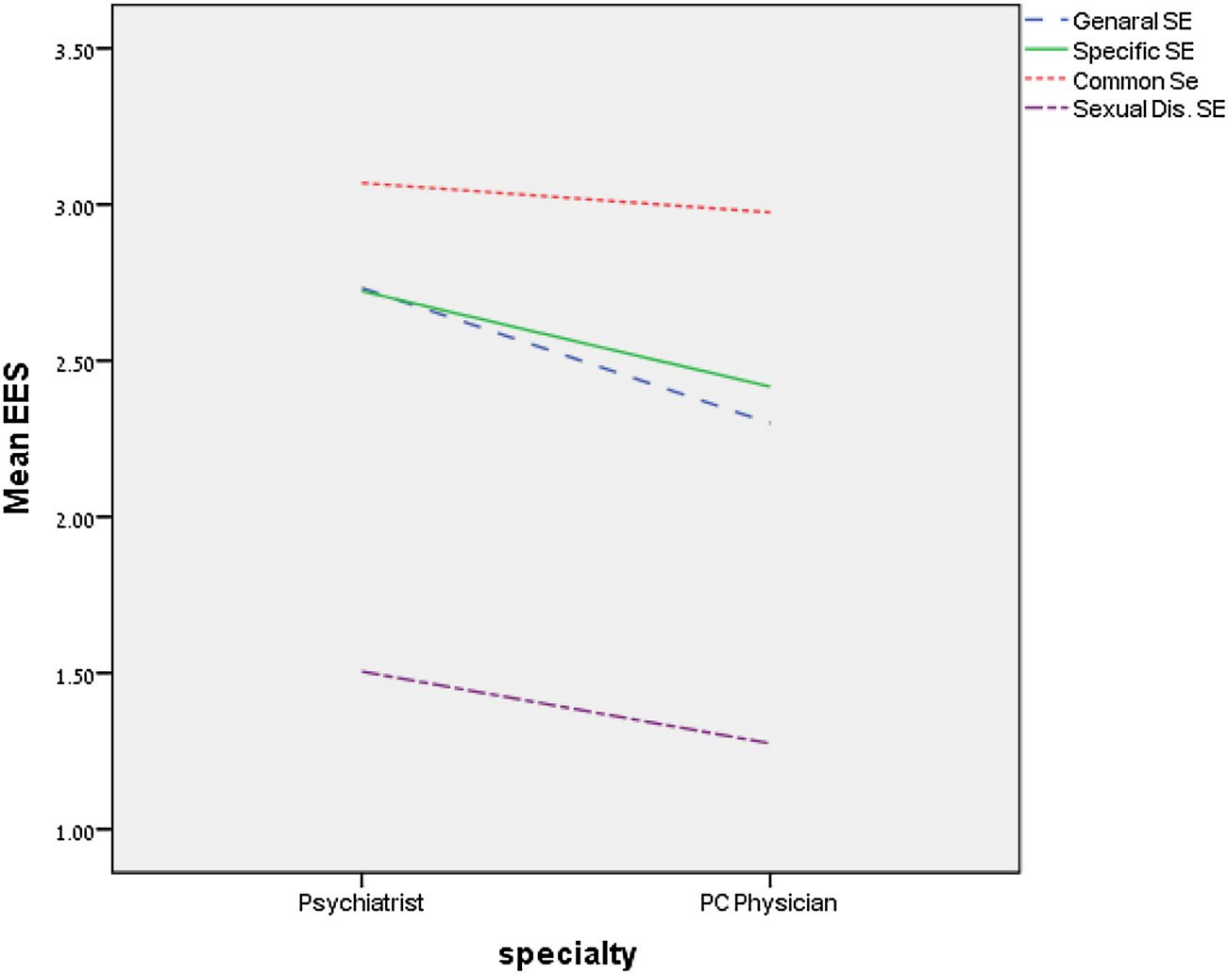
Multiple comparisons of SE’s discussion rates between Psychiatrists (*N* = 105) and PC Physicians (*N* = 123)

Further multiple comparisons (see Fig. 2) showed that psychiatrists address more SEs than do PC physicians (*t*(225) = 3.92; *p* < 0.001), as well as more often discuss the specific SEs (*t*(225) = 2.05; *p* < 0.05) and common SEs (*t*(225) = 2.01; *p* < 0.05) of SSRIs. Psychiatrists also referred to a larger variety of common SEs (*t*(225) = 2.47; *p* < 0.01). Finally, psychiatrists more often discussed the possibility of sexual dysfunction (*t*(225) = 2.23; *p* < 0.05), psychological SEs (*t*(225) = 3.02; *p* < 0.01), suicidal ideation (*t*(225) = 2.11; *p* < 0.05), and the mechanism of action of SSRIs (*t*(225) = 2.05; *p* < 0.05.)

Specialists initiated a discussion of the SEs of SSRIs more often than residents (*t*(225) = 2.04; *p* < 0.05), and more often discussed specific SEs (*t*(225) = 2.05; *p* < 0.05), sexual dysfunction (*t*(225) = 3.12; *p* < 0.01), and the mechanism of action of SSRIs (*t*(225) = 2.32; *p* < 0.05).

More specifically, PC specialists initiated discussion on the SEs of SSRIs more often than PC residents (*t*(225) = 2.10; *p* < 0.05). Senior psychiatrists also discussed SEs of SSRIs more often than psychiatry residents (*t*(225) = 2.02; *p* < 0.05), specifically the sexual SEs (*t*(225) = 4.01; *p* < 0.01) and the mechanism of action of SSRIs (*t*(225) = 2.51; *p* < 0.05). They also discuss more psychological SEs than residents (*t*(225) = 1.98; *p* < 0.05).

A greater patient workload correlated with a greater likelihood that the physician will discuss SEs in general (*r* = 0.25; *p* < 0.01) and severe SEs in particular (*r* = 0.20; *p* < 0.01), as well as sexual dysfunction (*r* = 0.16; *p* < 0.05), suicidal ideation (*r* = 0.16; *p* < 0.05), psychological SE’s (*r* = 0.29; *p* < 0.01), and the mechanism of action of the medicine (*r* = 0.16; *p* < 0.05).

Psychiatrists usually set follow-up appointments after 3 months (25 %), 1 month (46 %), or 1 week (12 %), while PC physicians mostly set the appointments after 6 months (21 %), 3 months (26 %), or 1 month (34 %). We also found that the more a physician discusses SEs with the patient, the greater the likelihood that they will both discuss strategies to deal with them later on during treatment and schedule an earlier follow-up appointment (*r* = 0.42; *p* < 0.01; *r* = 0.24; *p* < 0.01, respectively).

## Discussion

Our results indicate that many psychiatrists and PC physicians conduct an incomplete discussion on the SE profile of SSRIs with their patients. The extent of the discussion on SEs seems to be non-uniform among physicians, despite it being a central component of good clinical practice. This is surprising as shared decision making (SDM) is widely recognized as the preferred approach to doctor–patient interaction in clinical practice [16]. The application of SDM requires clinicians to share information as thoroughly as possible with their patients, and eventually reach a mutual consensus on treatment course. Emerging evidence suggests that SDM improves patient satisfaction with care, improves clinical outcome, and enhances adherence to treatment.

Our results suggest that discussing SEs is a clinical skill which positively correlates with other parameters of good clinical practice, such as scheduling an earlier follow-up meeting and discussing strategies to deal with SEs later on during treatment. Importantly, these clinical parameters overlap with guidelines for PC physicians regarding the treatment of depression [17, 18], emphasizing the role of explaining and evaluating SEs in good clinical practice.

Psychiatrists in our sample seem to discuss SEs more extensively than PC physicians. The assumption that specialty is a facet of expertise is supported by the finding that psychiatrists, as demonstrated in previous studies, seem to have more experience with, and extended pharmacological knowledge of, options in treating depression [19, 20].

The differences between psychiatrists and PC physicians in our study could not be explained by confounders such as gender or age of the physicians. A possible explanation of our finding is that psychiatrists deal with various psychopathologies (obsessive–compulsive disorder, panic disorder, etc.), greater severity and different types of depression (melancholic or psychotic VS dysthymia or atypical depression), and often have to differentiate depression from co-morbid physical illness. Their patients receive complex regimens and higher doses of SSRIs, and the psychiatrist is compelled to invest more time and attention in discussing SEs. However, this can hardly account for the difference between the groups, since the gap persists even when addressing SEs such as sexual dysfunction or GI symptoms that are equally common among patients treated with SSRIs by both psychiatrists and PC physicians [21]. Another explanation of the results might be that psychiatrists, compared with PC physicians, see their patients more frequently and spend more time with them at each meeting. However, this would not account for the differences between senior physicians and residents within each specialization.

Senior psychiatrists and PC physicians seem to explain and evaluate SEs of SSRIs to a greater extent than their respective specialty’s residents. It is important to note that in the Israeli medical system, senior PC physicians have at least the same patient workload as do residents. Therefore, expertise is a function not primarily of time spent with the patient, but rather of physician’s knowledge of SEs and length of time in practice, and the importance attributed to explaining and evaluating them.

Integrating proper management of SEs into educational interventions holds the potential of enhancing compliance and improving the level of care of depression in the community [22]. An interesting approach would be to view expertise in treating depression (indicated by specialty and seniority) as typified by a measurable difference in the quality of the discussion and evaluation of SEs. Previous studies have rarely attempted to quantify the quality of the explanation given by physicians about psychopharmacological treatments in general, and SEs in particular. Via the present survey, we introduce a simple questionnaire that enables health systems and physicians to evaluate this skill. Our results support the possibility of acquiring this facet of expertise throughout the course of time.

A study design based on a self-report questionnaire has all the limitations of this type of investigation. Furthermore, we did not check the test–retest reliability of the questionnaire. Though anonymous, the reliability of internet-based questionnaires can be questioned. However, it is important to note that results obtained over the internet did not significantly differ from those obtained through handouts. Another limitation of this study is that we lack data concerning the patients’ diagnoses and severity of illness. Finally, the study was held in a single geographical area and within the context of Israeli culture, and as such its findings could not definitively extend to physicians from other countries.

## Conclusions

It seems that PC physicians and psychiatrists do not share enough information on the SEs of SSRIs with their patients. Integrating proper management of SEs into educational interventions and clinical practice holds the potential of acquiring this facet of expertise throughout the course of time, and consequently enhancing compliance and improving level of care. In future longitudinal studies, the questionnaire-based scores, e.g., the EES, might be used for statistical prediction of treatment success (or failure), patient’s satisfaction, and adherence rates to antidepressant therapy.

## Abbreviations

PC: primary care
SEs: side effects
SSRIs: selective serotonin reuptake inhibitors
GI: gastrointestinal
EES: Expert Explanation Score
SDM: shared decision making
